# Technological and Digital Interventions for Mental Health and Wellbeing: An Overview of Systematic Reviews

**DOI:** 10.3389/fdgth.2021.754337

**Published:** 2021-12-23

**Authors:** Nele A. J. De Witte, Steven Joris, Eva Van Assche, Tom Van Daele

**Affiliations:** Expertise Unit Psychology, Technology & Society, Thomas More University of Applied Sciences, Antwerp, Belgium

**Keywords:** digital applications (apps), digital mental health, wellbeing, technologies, interventions

## Abstract

**Background:** Research increasingly shows how selective and targeted use of technology within care and welfare can have several advantages including improved quality of care and active user involvement.

**Purpose:** The current overview of reviews aims to summarize the research on the effectiveness of technology for mental health and wellbeing. The goal is to highlight and structure the diverse combinations of technologies and interventions used so far, rather than to summarize the effectiveness of singular approaches.

**Methods:** The current overview includes reviews published in the past five years with a focus on effectiveness of digital and technological interventions targeting mental health and wellbeing.

**Results:** A total of 246 reviews could be included. All reviews examined the effectiveness of digital and technological interventions in the context of care and welfare. A combination of two taxonomies was created through qualitative analysis, based on the retrieved interventions and technologies in the reviews. Review classification shows a predominance of reviews on psychotherapeutic interventions using computers and smartphones. It is furthermore shown that when smartphone applications as stand-alone technology are researched, the primary focus is on self-help, and that extended reality is the most researched emerging technology to date.

**Conclusion:** This overview of reviews shows that a wide range of interventions and technologies, with varying focus and target populations, have been studied in the field of care and wellbeing. The current overview of reviews is a first step to add structure to this rapidly changing field and may guide both researchers and clinicians in further exploring the evidence-base of particular approaches.

## Introduction

Within the broad field of healthcare and welfare a wide range of services are offered which are aimed at promoting the wellbeing and mental health of individuals. While the context and target populations can vary substantially, professionals in this field share many interventions which often rely on face-to-face interactions. However, digital technologies can also support these services, either stand-alone or in combination with an existing service offer. New technologies can allow for more flexibility, can offer interventions in the natural context, can reach a larger population without risk of stigma, and can be more cost-effective as compared to existing services (1, 2). Research increasingly shows how selective and targeted use of technology can have a meaningful impact on the quality of care and the role users can take in the organization and delivery of services (3). For example, users may be able to have more control over their care, especially in the context of chronic illness (4).

Nevertheless, there is a sharp contrast between what is technically possible and the amount of research that has actually been done so far. As a result, there are an overwhelming number of options, which hampers overview. To address this, attempts have already been made to structure parts of the field, for example for specific technologies, e.g., internet-supported mental health interventions (5, 6), smartphone apps (7) or for particular domains, e.g., for emotion regulation in clinical psychology (8). The current overview of reviews aims to extend those previous endeavors by expanding the scope to all technologies applied to the broad domain of mental health and wellbeing. The goal is to structure existing technologies and interventions which have been the focus of reviews, rather than to summarize the effectiveness of singular approaches. By summarizing the large body of research to date and by highlighting both similarities and differences across approaches and settings, we hope to further structure this domain and to inform about gaps in research that currently still exists.

## Materials and Methods

This review was preregistered in the Open Science Framework as part of a larger study (https://osf.io/hdxky).

### Search Strategy

The databases Scopus and Web of Sciences were searched on 4 January 2021 for reviews written in English and published in the past five years with a focus on effectiveness of digital and technological interventions in the field of care and welfare. A combination of two sets of search terms was used, one with a focus on technological interventions and the other on wellbeing. The search string was as follows: (websites OR “smartphone app^*^” OR wearable OR “virtual reality” OR “augmented reality” OR “immersive technology” OR platform OR mhealth OR “mobile health” OR ehealth OR “e-mental health” OR e-health OR internet OR mail OR chat) AND (“mental health^*^” OR “mental wellbeing” OR “social support” OR “psychological support” OR psycholog^*^ OR psychiatr^*^ OR “mental illness” OR “mental disorder” OR “quality of life”).

### Inclusion and Exclusion Criteria

Articles were included if they were systematic reviews, meta-analyses, scoping reviews, or overviews of reviews with an exclusive focus on the efficacy of technological tools or interventions in the context of mental health, wellbeing, or quality of life. No limitations were placed on the setting, control condition, or population, which could consist of participants of all ages of the general population, at-risk groups or individuals with underlying conditions. Studies were excluded if the focus was on strictly medical applications, lab research, assistive technology for disability, mere feasibility of technology, and if research took place among low- and middle-income countries.

### Literature Screening and Data Extraction

The online review platform Covidence (https://www.covidence.org) was used, which aims to facilitate screening and data extraction with multiple reviewers. Titles, abstracts and full texts were screened by two independent reviewers in each phase. Conflicts were resolved through discussion. A data extraction template was designed to extract the characteristics of each included study. Reviews were categorized regarding:

(1) Focus: prevention, treatment, or relapse prevention.
(2) Target population, or the intended audience of the reviewed interventions: general population, at-risk population, somatic disorders, pain or neurological disorders, substance use, mental illness, or other.
(3) Age: children and young adults, adults, or elderly.
(4) Setting: home, outpatient, or residential.
(5) Integration with conventional care: online, blended.

The included reviews were also labeled according to the intervention(s) and implemented technology(/ies) through inductive qualitative analysis with the goal of creating a taxonomy. Three authors independently developed an intervention and technology taxonomy based on 50 included reviews. These categorizations were subsequently compared, and the final matrix combining interventions and technologies was developed through discussion. All reviews were subsequently labeled according to these taxonomies. A review could load on multiple interventions and technologies simultaneously. After data extraction was complete, each entry was checked for errors in extraction by the first author.

For a number of combinations of interventions and technologies a review was selected and its focus described, to briefly illustrate what each combination might. Three criteria for selection were put forward: (1) the review is the most recent available, (2) the review is of high-quality, as determined by the JBI Critical Appraisal Checklist for Systematic Reviews and Research Syntheses [a maximum of four negative evaluations; (9)], and (3) the review focuses on the combination of a single methodology and technology.

## Results

### Study Identification

**Figure 1:**
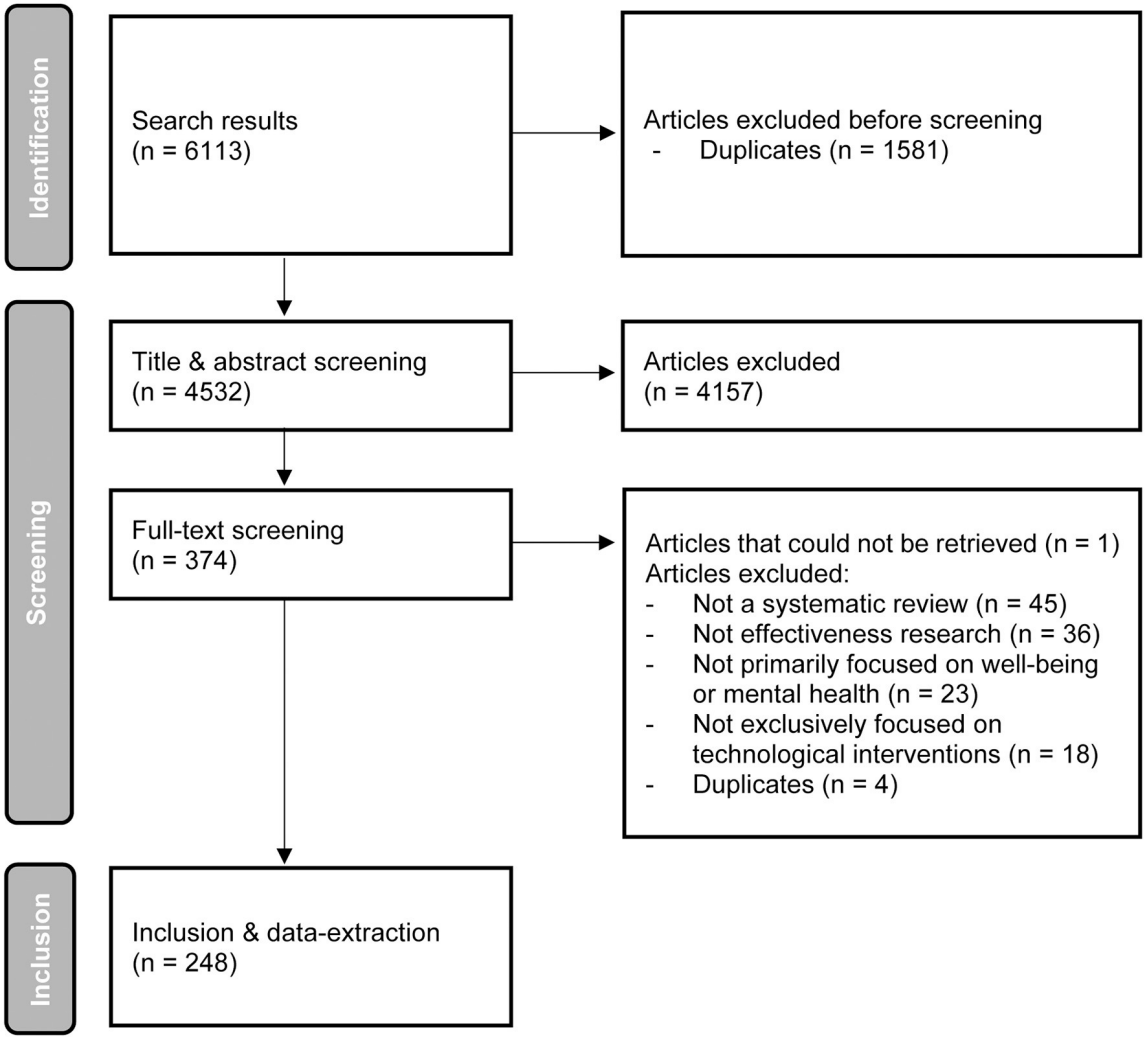
PRISMA flowchart.

The systematic search strategy yielded 6,113 results. The selection process is visually summarized in a PRISMA flowchart (10) in Figure 1. A total of 246 reviews could be included. All reviews examined the effectiveness of digital and technological interventions in the context of care and welfare. The reviews were diverse in scope and the quality of the studies they retrieved varied greatly (cfr. supra, review illustrations). Each review included an average of 16 studies, with outliers ranging from 1 to 111 studies.

### Characteristics of Reviews

**Figure 2:**
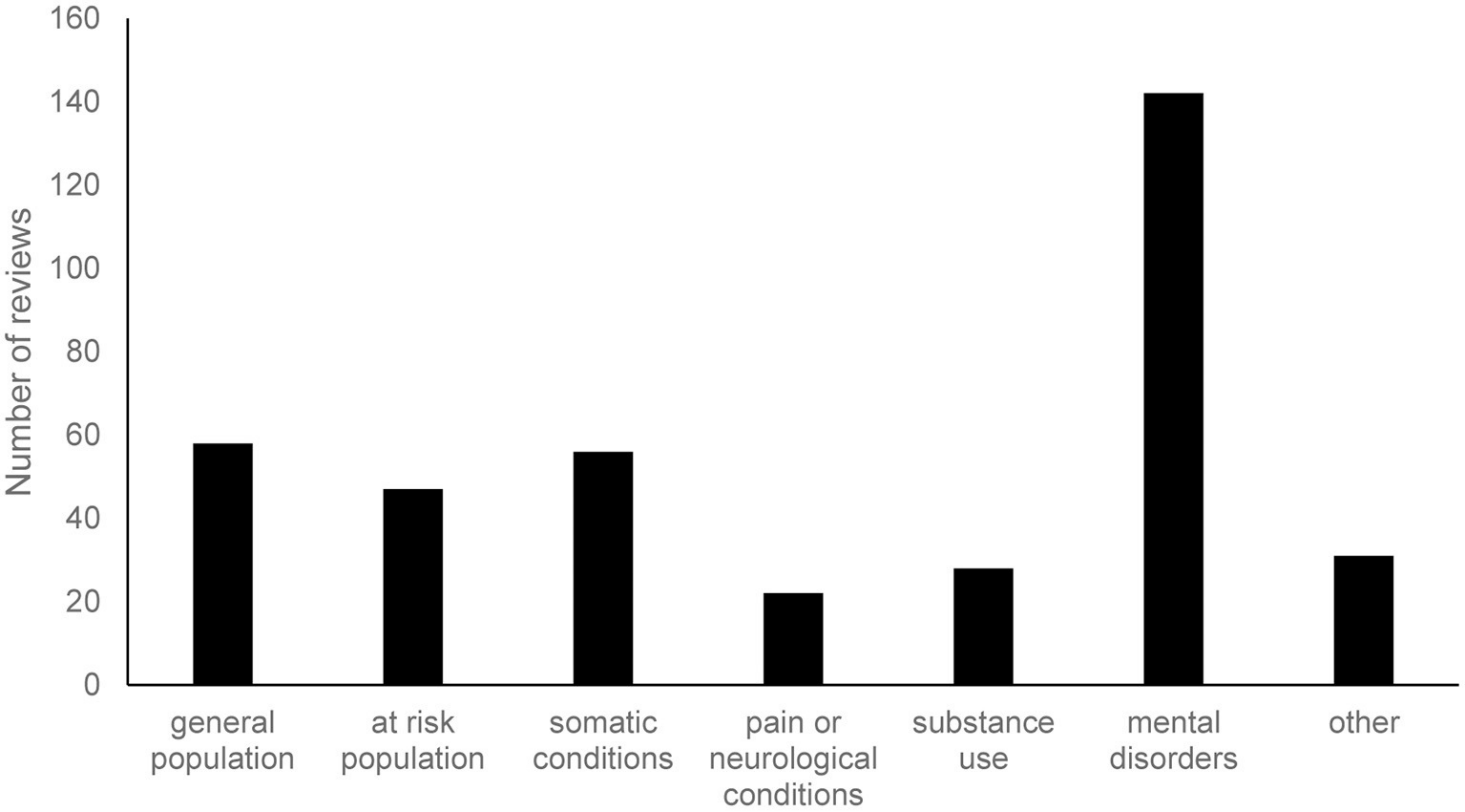
Target groups of the included reviews. Each review could be assigned multiple categories.

A detailed overview of the characteristics of each review can be found in Supplementary Table 1. Results show that most reviews focused on treatment (*N* = 196). Prevention was also fairly common (*N* = 107), but only a limited number of reviews examined applications for relapse prevention or maintenance of treatment effects (*N* = 20). In line with the focus on treatment or, in this context, psychotherapy, the largest subset of reviews had an (exclusive) focus on individuals with mental illness (*N* = 142; Figure 2). This was followed by the general population (*N* = 58) and somatic conditions (*N* = 56). The “other” category of Figure 2 consisted of informal caregivers (*N* = 22) and perinatal women (*N* = 9). A third of the studies focused on more than one target group in their review (e.g., both general population and at risk populations). In terms of age group, most studies focus primarily on adults (*N* = 203). However, there are also several studies that focus on children to young adults (*N* = 81). The age group that is currently understudied in systematic reviews is the older population (*N* = 23). Since most reviews do not clearly indicate the setting in which the studies took place (e.g., outpatient or residential), it was not possible to formally categorize reviews on this behalf. In general, however, many interventions were offered in the home setting. Additionally, interventions also took place in outpatient care, residential care, school/university setting, and the work context. Finally, there were only three reviews that clearly and explicitly examined blended care (a combination of online and face-to-face contact). Most reviews examined interventions that were entirely digital or did not clearly report whether complementary face-to-face contact was provided.

### Taxonomies

The taxonomy concerning interventions consists of 9 types, which are described in Table 1 and can be considered as an operationalization of the mental health intervention spectrum of mental disorders, developed by Mrazek and Haggerty (23).

**Table 1:** Taxonomy of interventions determined through inductive qualitative analysis, and their description.

**Intervention**	**Description**
Preventive interventions	Preventive interventions aim to prevent complaints and problems. Prevention can be aimed at the entire population (universal prevention), at individuals with increased risk (selective prevention), or at individuals who already have symptoms (indicated prevention) (11).
Measurement and follow-up	This includes all forms of measuring variables, whether by a professional, by the user themselves, or automatically. Therefore, this may involve (diagnostic) assessment, consisting of the measurement of the individual's strengths and needs through, e.g., questionnaires (12). In addition, it also involves self-tracking of one's own thoughts, behaviors, bodily sensations and/or emotions as they occur, which can be done through e.g., mood trackers (7). A final group of studies perform passive monitoring or data collection without any effort on the part of the user (13).
Supportive interventions	Interventions can be aimed at supporting the well-being of users. These interventions can use psychoeducation (i.e., providing information about disorders and their physical and mental consequences) which can for instance include a focus on compliance, management of disorders and coping with stress (14). In addition, activating and structurally involving the social network regularly plays a major role in this type of intervention.
Skills training	These interventions focus on mentally or actually practicing skills to build or strengthen a particular competency (12). This can for example consist of training social or cognitive skills. In addition, specific interventions such as biofeedback, i.e., supporting users to gain control over real-time physiological processes to improve their health and performance (15), can also be included.
Behavioral interventions	Interventions focusing on behavior change, trying to reduce risky behaviors or encouraging health-promoting behaviors (16, 17).
Gamified interventions	Interventions can also be offered with game elements. These are called “serious games” or games that actively engage the user and contribute to achieving a particular goal (18). While they are not merely entertaining, a user does not need to be aware of this. These games can for example be aimed at completing certain tasks, problem solving, cognitions, and promoting physical activity [exergames; (19)]
Psychotherapeutic interventions	The encyclopedia of Psychotherapy states that “Psychotherapy, defined within the broader context of the field of psychology, is a skilled and intentional treatment process whereby the thoughts, feelings, and behavior of a person are modified with the intention of facilitating increased functioning and life adjustment (20). Two streams of psychotherapeutic interventions were strongly represented in the literature and were therefore included as separate methodologies (see below).
Psychotherapeutic—Cognitive behavioral therapy (CBT)	CBT is a specific approach in psychotherapy that focuses primarily on the application of experimental methods and principles (such as learning principles) in clinical practice. Specific methodologies included within CBT are behavioral activation and exposure therapy. Behavioral activation consists of identifying, scheduling, and performing activities that are pleasurable or have a positive impact on mood with the goal of promoting or maintaining satisfying and enriching experiences (7). Exposure therapy for anxiety disorders consists of gradually exposing the user in a controlled manner to the stimuli and situations that evoke anxiety in order to reduce anxiety symptoms (21).
Psychotherapeutic—mindfulness & acceptance and commitment therapy (ACT)	Mindfulness and ACT, also called third wave CBT, are psychotherapeutic interventions focused on accepting difficult thoughts and emotions and encouraging behaviors that align with personal values (22).

Three broad clusters of technologies can be distinguished: a first is technologies taking conventional care online. A second is technology to be used as (stand-alone) interventions. Finally, there are emerging technologies, on which there may already be substantial research evidence, but which have not necessarily been (frequently) implemented in clinical practice up to now. Table 2 provides an overview of the taxonomy of technologies.

**Table 2:** Taxonomy of implemented technologies, determined through inductive qualitative analysis, and their description.

**Type of service**	**Technology**	**Description**
Conventional, but online	Synchronous media	Synchronous communication implies that the user and professional have real-time contact virtual contact via, for example, video calling or chat.
	Asynchronous media	In asynchronous communication, delayed exchange is expected, as is the case in e-mail conversations.
Programs	Computer or laptop	This consists for example of online platforms or installed software. It can exist in the form of self-help and in combination with support from a professional who (a)synchronously monitors progress and/or provides feedback.
	Smartphone	A second group of interventions is offered mobile via a smartphone application. It can exist in the form of self-help and in combination with support from a professional who (a)synchronously monitors progress and/or provides feedback.
	Digital interventions	This group covers a wide range of mixed digital interventions. In this lump category, reviews were placed that offered interventions that were both accessible via computer and smartphone and/or those that included peer support (e.g., via a forum) in addition to (a)synchronous follow-up from a professional.
Emerging technologies	Extended reality (XR)	Extended reality (XR) refers to virtual reality (VR), augmented reality (AR), and mixed reality (MR). VR refers to the experience of an immersive computer-simulated three-dimensional environment through a headset (24). AR refers to adding virtual elements to the real environment by means of the smartphone or a headset (21). MR involves a blending of the virtual and real worlds but is currently used very little.
	Social media	Social media (e.g., forums, social media platforms) can also lend itself to the provision of methodologies for care and well-being and are primarily used for peer contact and activating the social network.
	Wearables	This term refers to sensors and devices that can be worn on the body and can collect physiological and behavioral data (e.g., heart rhythm, physical activity) in a non-invasive manner continuously throughout daily life (25).
	Other	This includes chatbots, programs that can converse and interact with the user through spoken, written, and visual communication (26). In addition, robots, programmable machines that can perform tasks (semi) autonomously, can also be used for care and well-being. Another emerging technology is digital phenotyping or the use of automatically collected digital (usually smartphone) data to monitor functioning (27). Finally, game consoles and virtual classrooms (online educational spaces in which students and teachers interact) are also placed in this residual category.

Technological and digital interventions rely on the combination of a technical component and an intervention. The retrieved reviews can thereby be categorized under several interventions and/or technology forms. The matrix in Table 3 should therefore not be viewed as an exhaustive overview of possible combinations of interventions and technologies. What can be derived from it are common and less common combinations of technologies and interventions and the relative difference in weight of the various possible combinations.

**Table 3:** Overview of the number of studies retrieved in the reviews for combinations of interventions and technologies.

**Interventions**	**Technologies**									
	**Conventional, but online**		**Programs**			**Emerging technologies**				
	**Synchronous**	**Asynchronous**	**Computer**	**Smartphone**	**Digital**	**XR**	**Social media**	**Wearables**	**Other**	**Total**
Preventive interventions			102	28	289	49		15	15	489
Measurement and follow-up	22	9		43	130	69		57	45	375
Supportive interventions	39	15	107	14	659	39	49	56	51	1,029
Skills training			54	22		58			44	178
Behavioral interventions	13		21	58						92
Gamified interventions			30			20		5	17	72
Psychotherapeutic interventions	200	71	399	354	1372	531		221	256	3,350
Psychotherapeutic—CBT	19		520	8	244	15				806
Psychotherapeutic—mindfulness & ACT			97	61	157				33	348
Total	293	95	1330	588	2851	781	49	354	461	6,414

*ACT, acceptance and commitment therapy; CBT, cognitive behavioral therapy; XR, extended reality*.

The overview in Table 3 shows that the focal point of research into technological and digital interventions for mental health and wellbeing is on interventions offered through computer, smartphone, or a combination of both.

Programs can be offered in the form of mere self-help or can include some form of (mainly digital) support from a professional. Table 4 provides an overview of the number of studies included in the reviews that discuss pure self-help in relation to studies that (also) include interventions that are supported by a professional. While the majority of studies on smartphone applications are limited to self-help, studies exploring computerized programs do more commonly include professional support. Conventional services delivered online are less represented. In terms of emerging technologies, studies using VR and wearables are most common. When inspecting the interventions, and in line with the predominance of samples with mental disorders, psychotherapeutic interventions are strongly represented in the literature. Supportive interventions are also common and mostly target informal caregivers and individuals with somatic conditions.

**Table 4:** Overview of the number of studies retrieved in the reviews for combinations focusing on interventions and self-help.

**Interventions**	**Programs**			
	**Computer**	**Of which**	**Smartphone**	**Of which**
		**self-help**		**self-help**
Preventive interventions	102	20	28	13
Measurement and follow-up			43	43
Supportive interventions	107	8	14	14
Skills training	54	54	22	22
Behavioral interventions	21	13	58	58
Gamified interventions	30	20		
Psychotherapeutic interventions	399	90	354	279
Psychotherapeutic—CBT	520	10	8	8
Psychotherapeutic—mindfulness & ACT	97		61	34
Total	1330	215	588	471

*ACT, acceptance and commitment therapy; CBT, cognitive behavioral therapy*.

### Illustrations of Reviews Within the Combination of Both Taxonomies

Discussing each of the 246 studies would lead us to far. To nevertheless offer some insight into both taxonomies, we briefly describe the most common combinations of technologies and interventions. For that purpose, we choose the most frequent combinations within the three technological clusters defined earlier and selected the review meeting all three criteria defined earlier. The JBI quality assessments of all retrieved studies can be found in Supplementary Table 2.

#### Conventional Approach Using (a)Synchronous Technologies

Corry et al. (28) focused on *synchronous technology and supportive interventions*. They conducted a systematic review on telephone interventions delivered by healthcare professionals, for providing education and psychosocial support to informal caregivers of adults with diagnosed illnesses. The combination of *asynchronous technology and psychotherapeutic interventions* was reviewed by Senanayake et al. (29). In text messaging interventions for the management of depression, texts were being used for various purposes: therapeutic, motivational and supportive.

#### Programs Supported by Technology

*Digital programs* were explored in combination with a wide variety of interventions, for example with *preventive interventions* in the context of technology-enhanced youth suicide prevention and interventions (30). Others were combined with *measurement and follow-up*, using digital interventions for routine outcome monitoring (ROM) and measurement-based care (MBC), the routine use of outcome measurement to guide treatment decisions of patients receiving face-to-face psychotherapy (31). Leng et al. (32) furthermore focused on the potential of combing *digital programs and supportive interventions*, more specifically the use of digital interventions to support informal caregivers of people with dementia. Victorson et al. (33), finally, explored the combination with *psychotherapeutic interventions—ACT and mindfulness* when looking into technology-enabled mindfulness-based programs.

*Self-help smartphone programs* were either most commonly combined with *behavioral interventions* or with *psychotherapeutic interventions*. On the one hand, a systematic review by Milne-Ives et al. (34) focused for example on the effectiveness of smartphone apps for health behavior change, in physical activity, diet, drug and alcohol use, and mental health. On the other hand, Ilagan et al. (35) looked into psychotherapeutic interventions targeting borderline personality disorder (BDP) symptoms like anger, suicidality and self-harm, using smartphone apps. These apps were used to set up safety plans, to help patients track their mood, or to facilitate emotion regulation exercises.

*Computer programs* often looked into *psychotherapeutic interventions, both general or CBT*, but were also used in the context of *skills training*. Dugdale et al. (36) summarized current evidence on the potential of computer-based treatment programs to reduce symptoms of substance misuse and mental health difficulties in adults with a dual diagnosis. Following an initial screening, users could for example access an interactive coping strategy training, which helped them to address the lifestyle factors which are maintaining their harmful alcohol consumption. Eilert et al. (37) conducted a systematic review and meta-analysis on the effectiveness of computer-based treatment for generalized anxiety disorder. All but one of the online interventions included some form of human support alongside the intervention and most were primarily based on CBT. To be more specific, interventions relied on (a combination of) psychoeducation, case examples, mindfulness and/or relaxation exercises, notification and/or reminder emails, homework, summaries, and relapse prevention and maintenance. Finally, a scoping review by Zhang et al. (38) assessed the potential of computer-based cognitive bias modification interventions. These interventions, for which evidence initially emerged from experimental psychology, aim to retrain automatic attention to stimuli that are either harmful (e.g., in the context of substance abuse) or threatening (e.g., in the context of social anxiety disorder). In order to do so, participants for example completed online series of modified Stroop tasks. In these tasks, the computer presented them with series of threatening and neutral words, in varying colors. Every time, participants were asked to name the color of these words, while ignoring their semantic content.

#### Emerging Technologies

*XR* was most commonly used in combination with *psychotherapeutic interventions—CBT*. Kothgassner and Felnhofer (39) examined the effectiveness of virtual reality exposure therapy (VRET) for the treatment of anxiety disorders in children and adolescents. *Social media*, in turn was most frequently combined with *supportive interventions*. Ridout and Campbell (40) conducted a systematic review on the current evidence base for using social networking sites as a means to deliver mental health interventions for young people up to the age of 25, particularly for sharing knowledge and providing peer-to-peer support. *Wearables* were often used for *measurement and follow-up* and were for example used to unobtrusively measure and monitor depressive symptoms in children and adolescents (41). *Chatbots* were applied in the context of *pychotherapeutic interventions*, for example in the review by Abd-Alrazaq et al. (26) who explored to what extent chatbots might meet the needs of people with mental health conditions, in particular people with symptoms of depression, anxiety and stress and acrophobia. Emerging technologies which are less frequent in current literature are digital phenotyping and robots. Cornet and Holden (13) looked into the potential of *digital phenotyping* for health and wellbeing and found that smartphones were most commonly used to capture accelerometery, location, audio, and usage data, for example with patients with bipolar disorder or schizophrenia. The obtained data were primarily used for unobtrusive monitoring. Finally, Scoglio et al. (42) was the only review with an exclusive focus on *robots*, but found only a very limited number of studies to date.

## Discussion

The current overview of reviews on technological and digital interventions in the field of care and welfare shows that there is a large diversity, both in terms of interventions and technologies used. Although the focus is mainly on treatment, a relevant portion of the reviews also consider a preventive approach. Furthermore, both in the case of young people and adults, the reviews focus on a wide variety of target groups. No clear-cut differences regarding these target groups were found amongst the diversity of retrieved reviews, aside from the fact that supportive interventions mostly targeted informal caregivers and individuals with somatic conditions. Technologies most frequently researched are programs, both on computers, smartphones, or cross-platform digital environments (74% of all study categorizations). Not only emerging technologies (e.g., XR), but also technologies that allow conventional therapy to take place online (e.g., video calling) have been the focus of research far less often. Remarkably, an explicit focus on blended interventions is largely absent from the reviews. Combining online and face-to-face offerings is often cited as the most promising avenue for technological and digital interventions in care and welfare (43). However, only three of the 246 reviews appear to explicitly focus on this.

A classification matrix focusing on the type of technology and the content of the activity or intervention was created through qualitative analysis. This proved challenging as many reviews included a wide range of interventions. While the current solution can help to understand the variety of possible interventions and gaps in research and practice, other ways of classification are also possible and have been proposed. The list of treatment elements and definitions for the classification of smartphone apps by Wasil et al. (7) for example demonstrated that psychoeducation, relaxation and medication were the three most common elements in smartphone apps. Since our study goes beyond smartphone apps and focuses on reviews that each include a broad range of treatment elements, a categorization at a higher level was warranted. Fernandez-Álvarez et al. (8) recently made a similar attempt to structure current research on digital technologies for the intervention of emotion regulation, in which a distinction was made between three distinct categories: digital technologies (1) to understand process and outcome (e.g., (bio)sensors, (2) to create new interventions (e.g., bio- and neurofeedback and XR) and (3) to disseminate psychological treatments [e.g., (un)guided interventions and videoconferencing psychotherapy]. Although their categorization is solely focused on emotion regulation, the structuring does show similarities to the current overview of review. However, as our study took a more systematic approach and looked at combinations of technologies and interventions in a broader field, it might offer better insights in current gaps and potential opportunities. The potential of XR is for example not limited to creating new interventions, but might very well also be used to understand process and outcome. A structuring of the field using the combination of two taxonomies therefore seems to better allow for a flexible categorization of (future) studies.

The focus of future research can be twofold. Firstly, it can further explore novel combinations of interventions and technologies, as combining both taxonomies resulted in a 9 x 9 matrix. This implies that, theoretically, 81 combinations could be made of different technologies and interventions. However, no studies were retrieved for 31 of those combinations (38%). This might lead one to conclude that the different forms of technology have only been used rather one-sided so far. However, not every technology is necessarily suitable for every intervention. Nevertheless, there are various technologies included in the matrix that are still recent: research on these paradigms has therefore only recently started developing. Those emerging technologies have currently been relatively understudied, except for XR, where virtual reality already has an extensive and long-standing research tradition, but is only now gradually making its way into practice. Secondly, even more established combinations need further strengthening, especially those who already see strong uptake in clinical practice. To be more specific, this overview for example shows that (self-help) smartphone apps and programs, which are currently already frequently disseminated in practice, have not been the focus of as much research as often might be thought.

There are also limitations to the current study. Given the broad scope of our overview and the large number of reviews retrieved, we opted to rely on the count of studies within reviews as an indication for the amount of research that has been conducted to date. This obviously does not provide a proper indication of the actual evidence-base for particular combinations of technologies and interventions. We are therefore cautious in our interpretations and see the current overview primarily as a way to provide a structuring of a very broad field, which is currently still in the midst of expanding. Also important to note is that this overview was set up in the context of a broader study on the potential of technological and digital interventions for Flanders, a region and community in Belgium. To assure sufficient local relevance of the literature overview, it was therefore limited to only include reviews with a focus on high-income countries. In the initial abstract screening process several articles were excluded focusing on low to middle income countries. Digital interventions seem (also) most prevalent there as well, although the number of studies specifically taking these contexts into account are still limited (44).

Taken together, the current overview shows that technological and digital interventions in the field of care and welfare can vary substantially in terms of the aims for which they are used, their focus, and target population. Overall, reviews focusing on effectiveness of such applications do appear to have mostly concentrated on psychotherapeutic interventions for mental illness offered through computers and smartphones. Regardless of the underlying rationale, however, adding structure to this diverse and rapidly expanding field helps to offer some insights in current (lack) of evidence-base of certain technologies and interventions that rely on them.

## Author Contributions

EV, SJ, and ND created the taxonomy. All authors contributed to the screening process and participated in the data extraction and the writing of the manuscript.

## Funding

The funding for this overview was provided by the Policy Research Centre Welfare, Public Health and Family of the Flemish Government (Project EF73).

## Conflict of Interest

The authors declare that the research was conducted in the absence of any commercial or financial relationships that could be construed as a potential conflict of interest.

## Publisher's Note

All claims expressed in this article are solely those of the authors and do not necessarily represent those of their affiliated organizations, or those of the publisher, the editors and the reviewers. Any product that may be evaluated in this article, or claim that may be made by its manufacturer, is not guaranteed or endorsed by the publisher.
