# Supplementary material for: Technological and Digital Interventions for Mental Health and Wellbeing: An Overview of Systematic Reviews
Source: Front Digit Health. 2021 Dec 23;3:754337. doi: 10.3389/fdgth.2021.754337 (PMC8732948; doi:10.3389/fdgth.2021.754337)
Supplement: Supplementary file 2 [file Table_2.DOCX]

| Appendix 2  Review illustrations quality appraisal according to the JBI checklist | | | | | | | | | | | | | | | | | | | | | |
| --- | --- | --- | --- | --- | --- | --- | --- | --- | --- | --- | --- | --- | --- | --- | --- | --- | --- | --- | --- | --- | --- |
|  | | Conventional approach | |  | Programs | | | | | | | | |  | Emerging technologies | | | | | |  |
| JBI criterium | | Corry  et al.  (2019) | Senanayake et al. (2019) |  | Szlyk & Tan (2020) | Gual-Montolio et al. (2020) | Leng et al. (2020) | Victorson et al. (2020) | Milne-Ives et al. (2020) | Ilagan et al. (2020) | Dugdale et al. (2019) | Eilert et al. (2020) | Zhang et al. (2019)* |  | Koth gassner et al. (2020) | Ridout et al. (2018) | Sequeira et al. (2020)* | Abd-Alrazaq et al. (2020) | Cornet et al. (2018) | Scoglio et al. (2019) |  |
| 1 | Clear review  question | + | + |  | + | + | + | + | + | + | + | + | + |  | + | + | + | + | + | + |  |
| 2 | Appropriate inclusion criteria | + | + |  | + | + | + | + | + | + | + | + | + |  | + | + | + | + | + | + |  |
| 3 | Appropriate  search strategy | + | + |  | + | + | + | + | + | + | - | + | + |  | + | + | + | + | + | + |  |
| 4 | Adequate  (re)sources | + | + |  | + | + | + | + | + | + | + | + | + |  | + | + | + | + | + | + |  |
| 5 | Appropriate study  appraisal criteria | + | + |  | + | + | + | + | + | + | + | + | - |  | + | - | - | + | - | + |  |
| 6 | Appraisal by  ≥ 2 reviewers | + | + |  | + | + | + | + | - | + | + | + | - |  | + | - | - | + | - | + |  |
| 7 | Data extraction  minimised | + | + |  | + | + | + | + | + | + | + | + | + |  | - | - | - | + | - | + |  |
| 8 | Appropriate  study synthesis | + | + |  | + | + | + | + | + | + | + | + | + |  | + | + | + | + | + | + |  |
| 9 | Publication bias  assessed | - | - |  | - | - | + | + | - | + | - | + | - |  | - | - | - | + | - | - |  |
| 10 | Recommendations  supported by data | + | + |  | + | + | + | - | - | + | + | + | - |  | + | + | + | + | + | + |  |
| 11 | Appropriate directives  new research | - | - |  | - | + | + | + | + | + | + | + | + |  | + | + | + | + | + | + |  |
